# The different definitions of multimorbidity and their implications for research, surveillance, and policy

**DOI:** 10.1093/eurpub/ckae193

**Published:** 2024-12-02

**Authors:** Sarah Cuschieri, Saverio Stranges, Tatjana T Makovski

**Affiliations:** Faculty of Medicine and Surgery, University of Malta, Msida, Malta; Department of Epidemiology and Biostatistics, Schulich School of Medicine & Dentistry, Western University, London, Ontario, Canada; Department of Epidemiology and Biostatistics, Schulich School of Medicine & Dentistry, Western University, London, Ontario, Canada; Department of Family Medicine, Western University, London, Ontario, Canada; Department of Medicine, Western University, London, Ontario, Canada; Department of Clinical Medicine and Surgery, University of Naples Federico II, Naples, Italy; Department of Non-Communicable Diseases, French Public Health Agency (Santé Publique France), Saint-Maurice, France

The inaugural hybrid International Symposium, jointly organized by the EUPHA Chronic Disease Section and Santé Publique France, held on 26–27 June 2024, at Santé Publique France in Paris, marked a key shift in the dialogue from managing and preventing single chronic diseases to tackling the complexities of multimorbidity in both public health and primary care settings. Experts from across Europe explored multimorbidity’s prevention, surveillance, clinical management, and policy implications. This editorial aims to provide a synthesis of the symposium’s key messages on multimorbidity definitions and their potential impact on research, surveillance, and policy.

Multimorbidity is broadly defined as two or more chronic conditions coexisting in the same individual without prioritization, allowing flexibility but complicating comparability across studies. The first definition by van den Akker *et al*. [1] was later revisited by Boyd and Fortin [2] to emphasize the importance of selecting relevant chronic conditions, underscoring challenges in achieving consistency. Over time, different definitions have evolved such as the one provided by the National Institute for Health and Care Excellence (NICE) in the UK limiting multimorbidity to two or more long-term conditions—physical, mental, sensory, or chronic diseases—which helps streamline clinical focus. However, this approach may overlook acute conditions and biopsychosocial factors (biological, psychological, sociodemographic) that significantly influence health [3]. The Academy of Medical Science includes long-term infectious diseases, like hepatitis C and HIV as part of multimorbidity definition [4], highlighting how different healthcare settings address both chronic and long duration infectious conditions. Researchers also vary in their definitions. Some take a more holistic approach, incorporating acute conditions, biopsychosocial elements, and somatic risk factors to capture the complex dimensions of health beyond chronic diseases, especially useful in clinical settings where a comprehensive patient view is essential. A Delphi study [5], involving expert panels, recommended defining multimorbidity as two or more long-term conditions, including those that are active, permanent, require treatment or surveillance, or need ongoing management. The study noted that simple condition counts are useful for estimating prevalence, but weighted measures more accurately assess disease burden. However, consensus on defining “complex multimorbidity”—where multiple chronic conditions interact to significantly impact quality of life and healthcare needs—remains elusive, highlighting the challenges in capturing the full complexity of these cases [5].

The variability in multimorbidity definitions undermine international comparisons of prevalence estimates and obscures crucial disease patterns. Instead of mere counts, multimorbidity might be better understood as clusters of interrelated conditions, impacting outcomes differently. For example, it is well known that cardiometabolic clusters (e.g. diabetes, hypertension, obesity) often arise from shared lifestyle factors, while mental health issues like depression frequently co-occur with chronic pain, musculoskeletal, or cardiovascular conditions. Depression may greatly impact quality of life, while heart disease strains healthcare through hospital admissions. Recognizing such clusters incentivizes the organization of more comprehensive, rather than single-disease-centered, healthcare that better anticipates healthcare needs, costs, and patient outcomes. To improve comparability, researchers need more nuanced measures that account for disease interactions and severity, not just condition counts. Measures here include tools that assess multimorbidity complexity, like weighted indices and a comprehensive list of relevant conditions, ensuring more accurate findings and better support for health planning and resource allocation.

The lack of standardized multimorbidity definitions jeopardizes public health surveillance, which remains rare worldwide. Varying definitions yield inconsistent prevalence estimates depending on included conditions and criteria, making it difficult to monitor trends or allocate resources efficiently. Data quality and availability further complicate surveillance. Countries with advanced electronic health records can apply complex definitions, including biopsychosocial factors, while those with limited data may rely on simpler condition counts. Differences in data and disease prevalence hinder harmonizing global surveillance; however, high- and middle-income countries (focusing on chronic diseases) and low-income countries (managing both chronic and infectious diseases) may find some common ground.

The absence of a standardized definition poses obstacles for policymakers, as differing definitions fragment the evidence base, making it hard to address multimorbidity needs effectively. Broader definitions may amplify the perceived burden, justifying more healthcare funding, while narrower definitions could understate it, supporting budget cuts. This flexibility can skew policy discussions, leading to suboptimal strategies. Countries may approach multimorbidity differently based on definitions and perceived priorities, fragmenting strategies and limiting collaborative prevention and management efforts.

A single universal definition may be impractical, but distinct definitions for research, surveillance, or clinical practice—along with a minimal list of core diseases to be included—could improve consistency and relevance. Standardizing definitions and measurement tools, with transparent methodology and rationale, could foster coordinated global action on multimorbidity.

In conclusion, the diversity in multimorbidity definitions presents challenges across research, surveillance, and policy, complicating the accurate assessment of its burden on healthcare systems. Standardized definitions and transparent methodologies are needed to ensure consistent data collection and support international collaboration and policy initiatives to tackle more efficiently multimorbidity management and prevention.

Conflict of interest: None declared.
